# Immunotherapy for Metastatic Melanoma with Right Atrial Involvement in a Patient with Rheumatoid Arthritis

**DOI:** 10.1155/2017/8095601

**Published:** 2017-12-28

**Authors:** Zachary Benson, Sarah Gordon, Patricia Nicolato, Andrew Poklepovic

**Affiliations:** Virginia Commonwealth University, 1250 E. Marshall Street, Richmond, VA 23219, USA

## Abstract

Prognosis for metastatic melanoma has improved significantly with the use of immune checkpoint inhibitors. Given improvements in survival, aggressive surgical treatment may be considered in patients with life-threatening complications from their disease that would not otherwise be considered in advanced disease. Patients with preexisting autoimmune diseases or prior immune-related adverse events from therapy are largely excluded from clinical trials. Concerns exist that immunotherapy in these patients could worsen autoimmune disease or increase the risk of developing additional immune-related adverse events on therapy. We present a case of a patient with rheumatoid arthritis that presented with obstructive heart failure secondary to melanoma that had metastasized to the right atrium. After aggressive surgical resection to stabilize him from his life-threatening heart failure, he was treated with ipilimumab, which was stopped due to an immune-related adverse event. He was then started on pembrolizumab and had a durable response to therapy. Aggressive surgical treatment should be considered in patients with a cancer that may respond to immunotherapy. Furthermore, some patients with preexisting autoimmune disease may be safely treated with checkpoint inhibition therapy, and patients with a severe immune toxicity from one class may successfully be treated with an alternate class.

## 1. Background

Melanoma is an aggressive cutaneous malignancy that accounts for 1 to 2 percent of all cancer-related deaths annually [1]. If detected early, surgical excision often leads to cure. However, prognosis is much worse if the cancer metastasizes. Although melanoma is the most common malignancy to spread to the heart, it is rarely diagnosed antemortem. Autopsy studies have estimated that over half of all patients with metastatic melanoma have cardiac disease, but very few are diagnosed because they are asymptomatic [2]. There are little data regarding life expectancy in a patient with cardiac metastases, but in general survival has ranged from an estimated 5 to 11 months in patients with metastatic melanoma [1]. Recently, prognosis for metastatic melanoma has improved significantly with the use of immune checkpoint inhibitor therapy. Consideration of aggressive surgical procedures in patients with metastatic melanoma may be warranted in the era of immune checkpoint inhibitor therapy as surgery may temporize patients from life-threatening aspects of their disease, allowing time for immunotherapy to positively affect their survival.

Immune checkpoint inhibition therapy for metastatic melanoma has been shown to improve survival. Monoclonal antibodies targeting the cytotoxic T-lymphocyte antigen 4 (CTLA4) and programmed death-1 (PD-1) pathways inhibit downregulation of the immune system, thereby allowing an enhanced T-cell immune response. These pathways are essential regulators in immune tolerance tissue, and their inhibition could lead to a myriad of autoimmune conditions known as immune-related adverse events (irAEs). Patients with preexisting autoimmune diseases were excluded from clinical trials of these therapies, and only one trial included patients with a prior irAE [3]. Here, we present a case of a patient with rheumatoid arthritis that presented with heart failure secondary to cardiac melanoma with an unknown primary lesion. He was successfully treated with aggressive surgical resection and immune checkpoint inhibition.

## 2. Case Presentation

**Figure 1:**
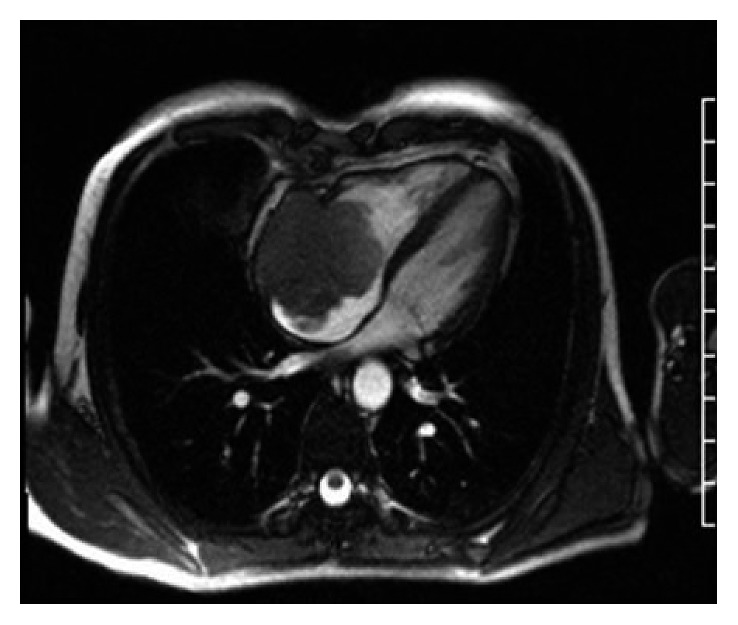
Cardiac MRI demonstrating 5.3 × 5.4 right atrial mass.

A 54-year-old white male with a past medical history of rheumatoid arthritis on anti-TNFalpha therapy with etanercept was admitted to the hospital with a 3-month history of dyspnea on exertion, fatigue, and lower extremity edema after a transthoracic echocardiogram (TTE) revealed a reduced ejection fraction of 40% with a large right atrial mass. Cardiac magnetic resonance imaging (MRI) identified a 5.4 × 5.3 centimeter lobulated right atrial mass (Figure 1) with extension through the right atrial wall and probable pericardial invasion. MRI of the abdomen and pelvis showed multiple hepatic lesions, and the largest measured was 6.6 × 7.0 × 7.3 centimeters. Abdominal MRI demonstrated mass effect from the hepatic lesions on the bile duct, hepatic portal veins, inferior vena cava, and the first portion of the duodenum. A liver lesion was biopsied, confirming melanoma, BRAF, and cKIT wild type. A primary cutaneous lesion was never identified.

**Figure 2:**
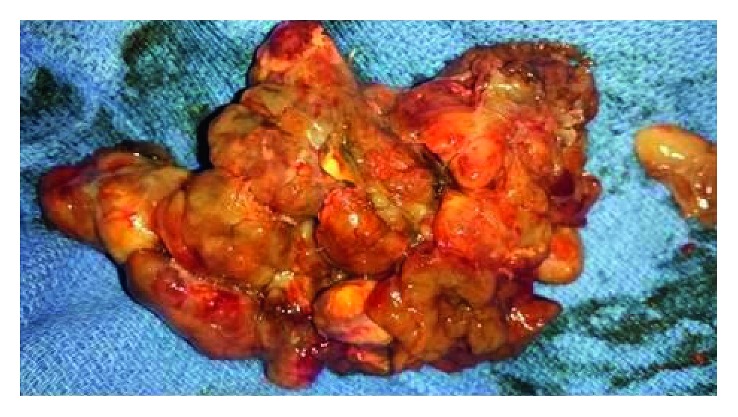
Photo of surgically excised right atrial mass.

The patient was stabilized and discharged with outpatient medical oncology follow-up to discuss treatment. However, days prior to his appointment he returned to the Emergency Department with worsening dyspnea due to the right atrial mass. Although he had not received treatment for his metastatic melanoma, heart failure due to obstructive cardiac metastasis is generally a poor prognostic indicator. Consequently, the benefits and risks of the procedure were extensively discussed between the medical oncologists and cardiothoracic surgeons. It was determined to proceed with aggressive measures, given the potential for long-term durable responses from immune checkpoint inhibitor therapy. He underwent a radical resection of the right atrial mass (Figure 2) and reconstruction with a pericardial patch. Following the procedure, a TTE showed normal cardiac chambers and improvement in his ejection fraction to 55–60%.

After recovery from surgical resection of the metastatic heart lesion, the patient was started on immunotherapy. The patient's rheumatoid arthritis was previously well controlled with etanercept monotherapy, which was stopped prior to treatment. He was started on 3 mg/kg dose of ipilimumab (anti-CTLA4) every 3 weeks. After 3 doses, he developed grade III acute kidney injury, nephrotic-range proteinuria, and anasarca requiring hospitalization. Renal biopsy demonstrated minimal change disease with acute interstitial nephritis. His creatinine increased to 5.22 mg/dl from a baseline of 1 mg/dl. He was treated with pulse methylprednisolone at 1 gm/day and intravenous diuretics with gradual improvement in his kidney function. He did not require hemodialysis, and his steroids were successfully tapered over the next 3 months. Following his steroid taper, imaging demonstrated tumor progression in his peritoneal cavity despite improvement in his liver lesions and no recurrence in his heart.

Given clear evidence of progression outside of the pseudoprogression window, he was started on 2 mg/kg pembrolizumab (anti-PD-1) every 3 weeks. He had an objective response to treatment with reduction in tumor burden in his liver and peritoneal cavity. Before treatment with pembrolizumab, the patient's rheumatoid arthritis was controlled with prednisone 5 mg daily. After pembrolizumab treatment, he began experiencing diffuse arthralgias. Although arthralgias are a known side effect of anti-PD-1 inhibitors, he experienced significant morning stiffness and wrist swelling, indicating that he was having a true flare of his autoimmune disease. He was started on hydroxychloroquine for symptom control. With low-dose prednisone and hydroxychloroquine, his synovitis remained stable and low grade. Despite concurrent ongoing treatment with hydroxychloroquine and low-dose prednisone, his disease continued to respond to immunotherapy. The patient was treated with pembrolizumab for approximately 19 months, and he is currently living nearly 3 years after diagnosis in clinical remission and off therapy. He had no recurrence of kidney injury on PD-1 monotherapy.

## 3. Discussion and Conclusions

While metastatic melanoma frequently involves the heart, it is rarely diagnosed because it is typically asymptomatic [2]. Patients that are symptomatic may present with a pericardial effusion, superior vena cava syndrome, tachycardia, arrhythmia, or signs of congestive heart failure as illustrated in our case [4]. A unique aspect of this case was the rare presentation of congestive heart failure secondary to metastatic melanoma without a known primary. While immunotherapy has changed the prognosis of metastatic melanoma and durable long-term responses can now be seen, it generally does not work immediately. Therefore, patients symptomatic from cardiac metastases may require adjunctive surgery [5]. In this case, the aggressive surgical resection played an important role in the patient's long-term survival and symptomatic relief. As the efficacy of systemic therapy continues to improve the prognosis of metastatic melanoma, adjunctive surgery may play a more integral role in the treatment of some patients.

Multiple studies have demonstrated the importance of the CTLA4 and PD-1 pathways in immune tolerance. Both play a critical role in immune regulation, and decreased gene expression is associated with an increased risk of autoimmune disease [6–9]. Patients with preexisting autoimmune diseases pose an interesting challenge for clinicians. Concerns that anti-CTLA4 or PD-1 therapy would worsen their autoimmune disease or place them at an increased risk of developing irAE are certainly valid. Because patients with preexisting autoimmune diseases were excluded from initial clinical trials, each patient subsequently treated with these therapies adds to our understanding. Furthermore, only one published clinical trial with anti-PD-1 inhibitors included patients with a prior irAE with a CTLA4 inhibitor [3].

Our patient's durable response to anti-PD-1 therapy with minimal side effects is impressive given his history of rheumatoid arthritis and prior high-grade irAE. Successful treatment of a patient with anti-PD-1 therapy following high-grade irAE from ipilimumab therapy, without recurrence of that irAE, suggests that it may be possible for patients to be safely treated with immune checkpoint inhibitors of an alternate class following resolution of immune toxicity from the first agent.

A recent retrospective study evaluated the safety of anti-PD-1 use in 119 patients with either a preexisting autoimmune disease or an irAE related to prior ipilimumab treatment [3]. 38% of the patients with a preexisting autoimmune disease in this cohort had a flare of their disease requiring immunosuppression, but only 4% stopped treatment due to the flare. The patients with preexisting autoimmune conditions developed irAEs at a rate similar to the patients in the clinical trials [10, 11]. This is in contrast to patients with autoimmune conditions who were treated with ipilimumab and experienced irAEs more frequently than those without preexisting autoimmune disease [12]. The study found that reoccurrence of the same irAE secondary to ipilimumab in patients who had prior irAE was rare. However, new irAEs in these patients were often high grade and resulted in discontinuation of therapy. As was seen with our patient, the results from this study suggest that anti-PD-1 inhibitors are safe in patients with an autoimmune disease or a prior irAE from anti-CTLA4.

Our patient's clinical course is consistent with the emerging evidence that anti-PD-1 therapy is less toxic and yields better outcomes in comparison to anti-CTLA4 therapy [13]. Despite a minor flare of his rheumatoid arthritis requiring initiation of hydroxychloroquine, he has had an excellent response to immunotherapy after extensive cardiac surgery for his metastatic melanoma and is still alive nearly 3 years later.

## Abbreviations

CTLA4: Cytotoxic T-lymphocyte associated protein 4
irAE: Immune-related adverse event
MRI: Magnetic resonance imaging
PD-1: Programmed cell death
TTE: Transthoracic echocardiogram.

## Supplementary Materials

Supplementary Materials Left heart catheterization demonstrating neoangiogenesis of right atrial mass.
